# Supplementary figures and images for: Violence in fishing, hunting, and gathering societies of the Atacama Desert coast: A long-term perspective (10,000 BP—AD 1450)
Source: PLoS One. 2023 Sep 20;18(9):e0290690. doi: 10.1371/journal.pone.0290690 (PMC10511140; doi:10.1371/journal.pone.0290690)

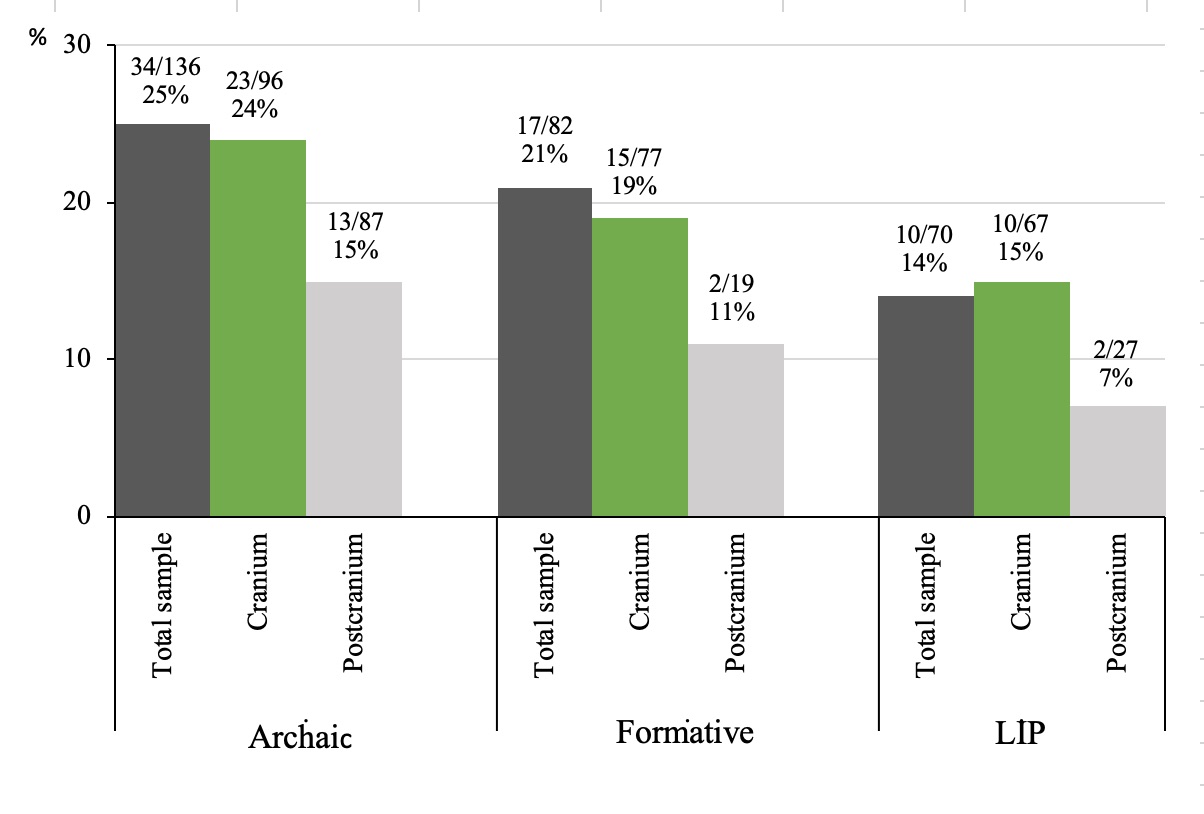

Supplement: S1 Fig — Archaic, Formative, and Late Intermediate Periods: gray bar, considers the total N of the sample regardless of the degree of completeness. (JPG) [file pone.0290690.s002.jpg]

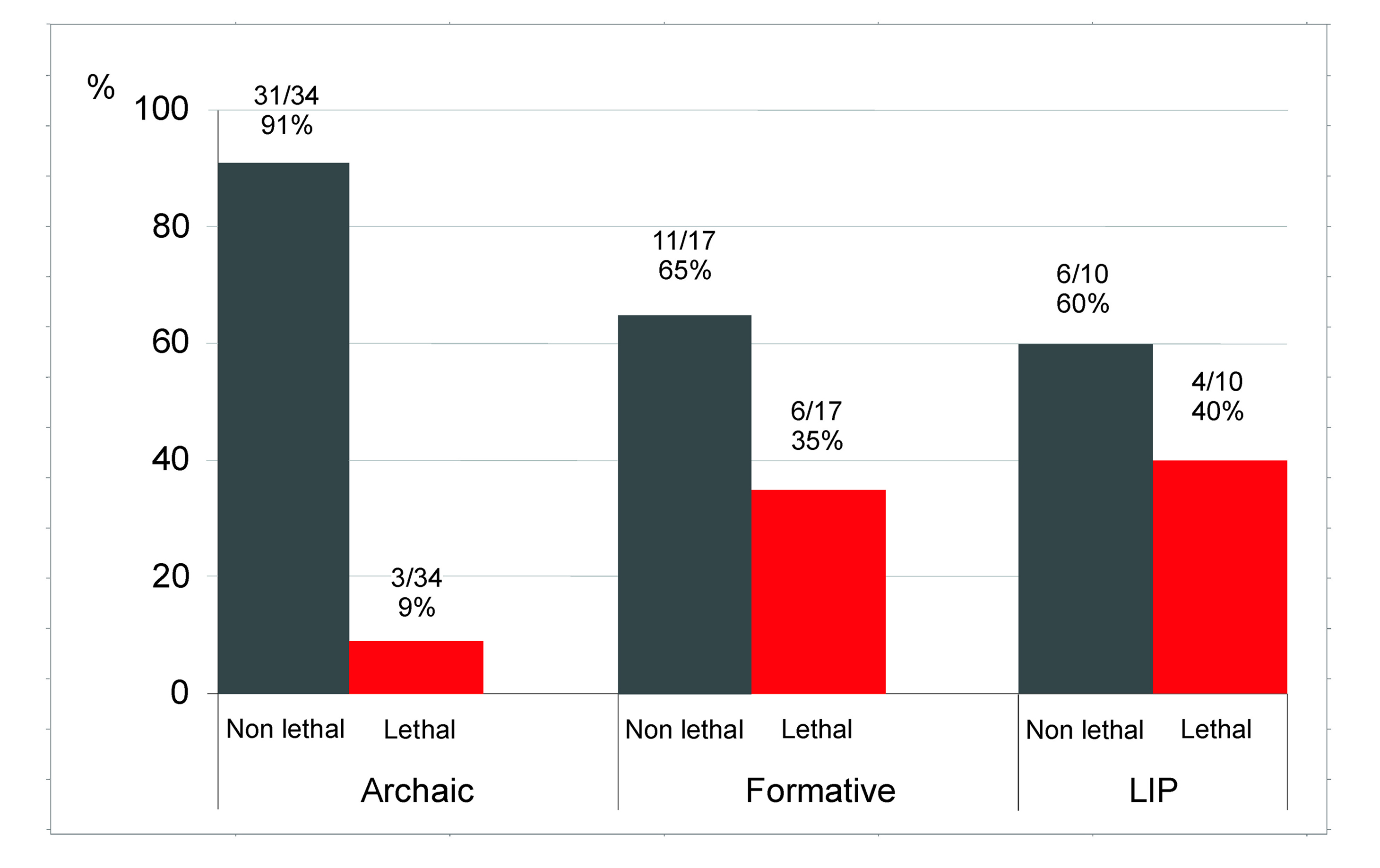

Supplement: S2 Fig — Archaic, Formative, and Late Intermediate Periods. (JPG) [file pone.0290690.s003.JPG]
